# The Registration of Nurses

**Published:** 1889-12-21

**Authors:** 


### The Hospital

A Weekly Journal of

**Science, Medicine, Nursing, and Philanthropy.**

*For Hospitals, Asylums, Medical Practitioners, Students, Nurses, Families, Parishes, Congregations, and the Charitable Public.*

Vol. VII. No. 169.] [December 21, 1889.

### The Registration of Nurses.

Last week we dealt with the registration of nurses and midwives, a combination strenuously advocated by the British Nurses' Association, but which was rejected as impracticable by the General Council of Medical Education on the 29th ult. The views we have always advocated having been thus endorsed by the highest authority, and the registration of midwives being now provided for by Mr. Pease's Bill, drafted under the auspices of the Midwive's Institute, the registration scheme put forward by the British Nurses' Association remains for consideration. Like other astute persons, the leaders of the British Nurses' Association have been very careful not to publicly formulate any definite plan. So much of their scheme as was embodied in the draft charter has never been made public. For several months something practical has been promised, but has so far not been forthcoming. In these circumstances we have thought it right to publish in full the memorial of the British Nurses' Association to the Medical Council, and it will be found in another column of our present issue.

The General Medical Council had this memorial under consideration on the 29th and 30th of November. On the 29th, the British Nurses' Association's proposal to appoint a registration board for nurses and midwives, as embodied in paragraphs 1 to 4 of the memorial, was rejected. On the 30th, the remaining paragraph (5) of the memorial was considered by the council. This paragraph is as follows:

"(5.) The cardinal principle for which the British Nurses' Association has striven in the face of enormous opposition, is that the control of midwives and nurses should remain in the hands of the medical and nursing professions, and that this question of registration should be dealt with by professional authorities themselves. The association therefore submits this scheme to the General Medical Council, and respectfully seeks the advice, support, and assistance of this influential body in the matter. It would venture especially to ask whether it would be advisable that a certain number of representative medical men, obstetric physicians, and midwives should be requested to act as members of the Registration Council, and *ex officio* of the board. And, if so, whether in the opinion of the General Medical Council it would be better that these members should be elected by the Registration Council, or that they should be appointed by some outside authority. Finally, if the latter course be considered the more advisable, would the General Medical Council be willing to undertake the duty of nominating such representatives, as such action upon its part would be gladly accepted?"

On the 30th ult., on the motion of Sir John Simon, seconded by Dr. MacAlister, after the withdrawal of a definite resolution moved by Mr. Carter, it was resolved:

"That, in the opinion of the council, it would be much to the advantage of the public, and particularly would be of much convenience to the practitioners of medicine and surgery, that facilities, usable under proper guarantees in all parts of the United Kingdom, should be given by Act of Parliament or otherwise for the authoritative certification of competent trained nurses, who, when certified, should be subject to common rules of discipline, *but that it does not appear to be within the province of the council to propose legislation or other action for the object referred to, nor has the council had any occasion to consider in detail the means by which the object might best be attained, and that, under these circumstances, the council cannot give any opinion on the particular questions asked of it in paragraph 5 of the memorandum of the British Nurses' Association.*"

In again declining to identify itself with the scheme of the British Nurses' Association, the General Medical Council contented itself with the expression of a pious opinion, which briefly amounts to this: The Council has every sympathy with nurses and nursing, but it is a Council of Medical Education and Registration, the question of nurses being altogether beyond its scope and work. Hence the resolution merely states, in effect, that it would be advantageous to the public and the medical profession that facilities for the authoritative certification of competent trained nurses should be afforded in all parts of the United Kingdom. The council has, however, had no opportunity to consider in detail the means by which this object may best be attained, and cannot, therefore, give any opinion on the proposals embodied in paragraph 5 of the memorial of the British Nurses' Association. Should the Medical Council ever have occasion to consider in detail the means by which the registration of nurses can be best attained, that body will undoubtedly agree with the authorities of the nurse training schools and other representative persons that the certificates issued by those schools do already afford proper guarantees of competency in all parts of the United Kingdom. This being so, the resolution of the Council practically confirms the wisdom of the Signatories to the Memorial of the Nurse Training Schools. It is thus demonstrated that the promoters of the British Nurses' Association do not possess the requisite *locus standi*, and so are not practically competent to undertake the work of registration. It further follows that their whole movement is, as the nurse training school authorities contend, premature and unnecessary. Consequently, it is easy to understand why the organ of the leaders of the British Nurses' Association should have omitted to publish exactly that half of the resolution of the Medical Council which we have printed in italics, and so failed to give its readers an opportunity of realising the true meaning of the resolution passed by the Medical Council. Any comment on the honesty of such journalistic methods is entirely superfluous.

We have always stood by the nurses—that is, by every woman who has qualified herself to adequately discharge the whole duties of a nurse. It is gratifying to know that we have a larger number of nurses amongst our readers than any other journal published. We would therefore venture to ask each nurse to calmly consider, in view of the decisions of the Medical Council, whether she had not better, in homely phrase, "keep her money in her own pocket," at any rate for the present and rest content with the certificate she has received from the hospital where she was trained. If certificated nurses have any grievances or difficulties they should now be frankly stated, in order that they may be removed without delay.

Finally, it is well to remember that considerable misapprehension prevails as to the meaning of the word "registration." It has been constantly affirmed that a scheme of registration similar to that propounded by the British Nurses' Association would raise the status of nursing because the registration of medical practitioners by the Medical Council has had this result upon practitioners as a body. It is forgotten, or not understood by those who hold this view, that the Medical Council is endowed by law with penal powers, and that it can use them not only in dealing with individual medical men, but against the licensing and qualifying bodies who conduct the examinations for admittance into the medical profession. Thus the Medical Council can move the Privy Council to issue an injunction against any of the examining colleges or boards directing them to desist from certain practices, or it may proceed to revoke the power exercised by any such college or body to grant qualifications conferring the right to be registered. A voluntary board of registration for nurses would necessarily not possess any such powers or authority, or in fact any powers at all; nor could it exercise any control whatever over the authorities of the nurse training schools or over anyone else who at present grants certificates to trained nurses after examination. All that such a voluntary board could do would be to admit to their register, on payment of a fee, nurses who do not at present possess any certificate of training; or, in other words, it could do on a large scale and at enormous cost to nurses what an ordinary Servants' Registry does for servants on a small scale and at a small cost. But do trained and educated nurses desire to be connected with an overgrown Servants Registry, and to pay a very high price for the privilege?

Certificates issued under the British Nursing Association's auspices could be of no possible service to nurses at present holding certificates from nurse training schools, nor would they protect the public, but rather the reverse. The improvement which has taken place in the status, character, and training of nurses is mainly due to the action of the nurse training schools which now grant certificates; and any member of the public who employs a nurse can ask her to produce her certificate before engaging her. Should she not give satisfaction, or be guilty of misconduct, her employer can write to the authorities of the hospital who granted it. There can be no doubt that if this fact could be made generally known to the many well-intentioned people who have been bitten by the idea that registration would be an absolute protection to the public and an advantage to the nurse, the whole agitation would cease. The public must soon realise that registration is already a fact, and that a mere public registry office would add nothing to the nurse's status, whilst mulcting her in a heavy annual fee. The only point not yet compassed by the nurse training schools relates to a certain number of excellent women who have had ample experience and training in the calling of a nurse, but who are without certificates because they acquired both at small hospitals where neither examinations nor certificates are known. This point is brought forward by Miss Heanley in another column, and is under the consideration of the authorities of the nurse training schools at the present time. We have every reason to expect that steps will be taken by the nurse training school authorities to help these deserving and capable women to acquire certificates without difficulty or expense.

